# XON9—A Glyco-Humanized Polyclonal Antibody Effective Against Hepatocellular Carcinoma

**DOI:** 10.3390/ijms26189185

**Published:** 2025-09-20

**Authors:** Pierre-Joseph Royer, Carine Ciron, Gwenaelle Evanno, Ophélie Dauphouy, Juliette Rousse, George Graur, Odile Duvaux, Firas Bassissi

**Affiliations:** Xenothera, 44200 Nantes, France; carine.ciron@xenothera.com (C.C.); gwenaelle.evanno@xenothera.com (G.E.); ophelie.dauphouy@xenothera.com (O.D.); george.graur@xenothera.com (G.G.); odile.duvaux@xenothera.com (O.D.); firas.bassissi@xenothera.com (F.B.)

**Keywords:** hepatocellular carcinoma, immunotherapy, glyco-humanized polyclonal antibody

## Abstract

Hepatocellular carcinoma (HCC) is the main leading cause of cancer-related deaths. Treatments for advanced HCC include multikinase inhibitors (Sorafenib or Lenvatinib), with limited response rates and serious side effects, or immunotherapy applicable to a small fraction of patients. Thus, new strategies are needed to improve the management of HCC. We evaluate here the efficacy and safety of XON9, a first-in-class glyco-humanized polyclonal antibody (GH-pAb). Cytotoxic activity of XON9 against Hep3B, Huh7, HepG2 or primary hepatocytes was investigated. Apoptosis, caspase activity, production of reactive oxygen species (ROS) and mitochondrial membrane potential (MMP) were evaluated. Efficacy of XON9 was then assessed in vivo in NMRI nude mice, while pharmacokinetics and safety were evaluated in a non-human primate. XON9 showed a potent complement-dependent cytotoxicity (CDC) against Hep3B and Huh7 (EC50 < 10 µg/mL), and to a less extent against HepG2. XON9 induced apoptosis of HCC cells with activation of caspases 8 and 9, increase in ROS and drop in MMP. Overall, in vitro lytic activity of XON9 was superior to that of Sorafenib. In vivo, XON9 significantly reduced tumor progression and outperformed Sorafenib. No toxicity was observed after repeated injections of XON9 in a non-human primate. XON9 represents a promising and selective immunotherapy against refractory HCC.

## 1. Introduction

Hepatocellular carcinoma (HCC) is the most common primary liver cancer, representing 75% of the cases. It was one of the most frequently diagnosed cancer in 2024 and represents the third leading cause of cancer-related deaths worldwide [1,2]. Multiple risk factors for HCC include hepatitis B (HBV) or hepatitis C (HCV) infections, toxic exposure (alcohol, aflatoxins) or metabolite disorders (diabetes, obesity, non-alcoholic steatohepatitis). Vaccination campaigns and antiviral treatments have reduced virus-associated HCC [3]. Yet, the incidence of HCC is rising overall, in particular in the Western world, due to alcohol consumption and increase in metabolite disorders. Thus, in 2040, 1.4 million people are expected to be diagnosed with liver cancer, with a predicted death toll that could reach 1.3 million [4,5].

Management and prognosis of HCC greatly depend on the stage of the disease. Surgical resection applies for early-stage HCC and gives very good prognosis with median overall survival (OS) >10 years. Unfortunately, for most patients, HCC is diagnosed late, with a dramatic impact on the prognosis. For intermediate-stage HCC, conventional treatments such as transarterial chemoemolization (TACE) or transarterial radioembolization (TARE) provide a median OS of approximately 30 months [4]. The introduction of multikinase inhibitors (Sorafenib and Lenvatinib) has improved the management of unresectable advanced-stage HCC, yet the prognosis remains dismal with a median OS less than 15 months [6]. Immunotherapy with monoclonal antibodies (mAb) targeting immune checkpoint inhibitors (ICIs) or growth factors emerges as a potent therapeutic strategy for advanced-stage HCC, or for patients with intermediate stage-HCC showing disease progression under conventional treatments. Treatment with anti-programmed death-ligan 1 (PDL-1) plus anti-cytotoxic T-lymphocyte associated protein 4 (CTLA4) (Durvalimab plus Tremelimumab) has shown superiority over Sorafenib in improving OS in patients with unresectable HCC [7]. Furthermore, combination of ICI with anti-vascular endothelial growth factor (VEGF) (Atezolizumab plus Bevacizumab) provides significant benefits on OS and progression-free survival [8]. The association between ICI and multikinase inhibitors was investigated as well, with mitigated results on disease progression and patient survival [9,10]. Innovative strategies such as chimeric antigen receptor (CAR)-T cells that have shown remarkable efficiency in liquid tumors still face toxicity issues in HCC [11,12]. Moreover, as they target a limited number of antigens, they hardly mirror tumor heterogeneity and like mAb, they are susceptible to tumor escape [13].

Thus, despite this recent progress, the OS of patients with advanced-stage HCC remains low (<20 months) and less than 30% of patients show an objective response rate [4]. New treatments and strategies are thus needed to improve the clinical management of HCC. By targeting multiple epitopes, polyclonal multispecific antibodies (pAb) may be applicable to most patients, while minimizing the possibility of immune evasion. Although the antitumoral activity of pAb has been known for a long time [14], their clinical development has been hindered by their immunogenicity, due to the presence of xenogeneic patterns, mainly N-glycolylneuraminic acid (Neu5Gc) and α1,3 galactose (α-Gal) [15]. Low immunogenic glyco-humanized (GH)-pAb produced in genetically modified pigs devoid of Neu5Gc and α-Gal might renew the therapeutic potential of pAb in oncology [16]. GH-pAb have shown promising therapeutic proficiency in clinics, in infectious diseases [17,18] or in the setting of transplantation [19]. Their potential as anti-tumor treatment in HCC should be confirmed and compared to standard of care.

We evaluated here the therapeutic potential of XON9 first-in-class GH-pAb targeting HCC. The cytotoxic activity of XON9 in vitro was investigated and compared to Sorafenib in 2D-cultures or in 3D-spheroid models. The antitumor efficacy was then confirmed in vivo in a NMRI nude mice model, and safety was assessed in a non-human primate.

## 2. Results

### 2.1. Cytotoxic Activity of XON9 Against Hepatocarcinoma Cell Lines

**Figure 1:**
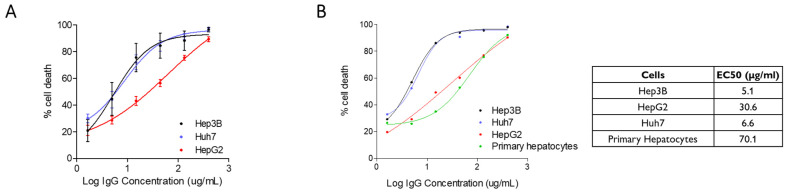
XON9 CDC activity. (**A**). Comparison of XON9 CDC activity against three HCC cell lines (*n* = 2). (**B**) Comparison of XON9 CDC activity against HCC cell lines vs. primary hepatocytes (*n* = 1). EC50 values are mentioned in the table.

We first measured the CDC activity of XON9 against HCC cell lines. Hep3B, Huh7 and HepG2 cells were cultured for 90 min with increasing concentrations of XON9 (1.65 µg/mL to 400 µg/mL) in the presence of rabbit complement. Strong and fast lysis of Hep3b and Huh7 cells was observed, with about 90% of cell lysis obtained with 100 µg/mL of XON9 after 90 min (Figure 1A). The lysis of HepG2 cell line was lower with 70% of cell lysis at 100 µg/mL. XON9 was also reactive against other tumor cells such as HCT116 (colon) or A549 (lung) but not CAPAN (pancreas) (Supplementary Figure S1) suggesting cross reactivity against tumor cells from various origins. XON9 binding to the three HCC cell lines was similar (Supplementary Figure S2), suggesting a difference in cell resistance to CDC rather than cell recognition by XON9. Yet, we found no major differences in the recruitment of C1q on HCC cells (Supplementary Figure S3A), nor in the expression of the complement regulators CD46, CD55 or CD59 (Supplementary Figure S3B). In addition, we did not detect any shedding of CD46. Blockade of CD46 and CD59 slightly increased XON9 activity on HepG2 cells (Supplementary Figure S4). The CDC activity of XON9 against HCC or primary hepatocytes was then compared (Figure 1B). Hep3B and Huh7 presented a higher sensitivity to XON9 compared to primary hepatocytes with a half maximal effective concentration (EC50) of 6.6 µg/mL and 5.1 µg/mL for Hep3b and Huh7 vs. 70.1 µg/mL for the primary hepatocytes. The activity of XON9 against HepG2 was slightly higher than against primary hepatocytes.

### 2.2. Activation of Apoptosis in HCC Cell Lines

**Figure 2:**
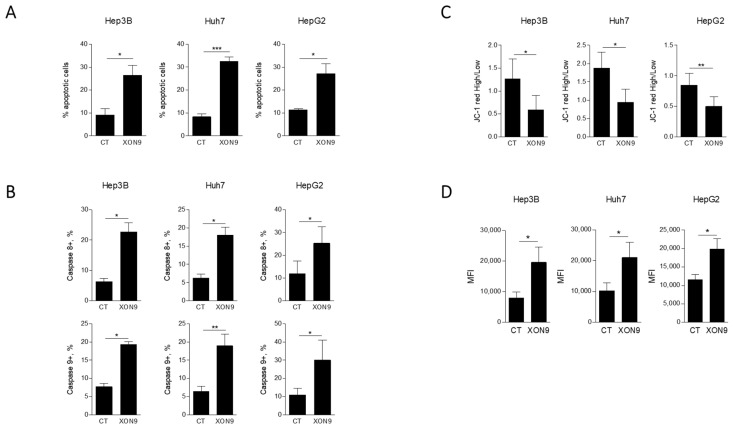
Investigation of apoptosis induction by XON9. Percentage of apoptotic cells after XON9 treatment (300 µg/mL, 24 h) (**A**). Percentages of HCC cells positive for caspases 8 or 9 (**B**). Mitochondrial membrane potential in HCC cells after XON9 exposure measured with the JC-1 probe (**C**). Levels of mitochondrial superoxydes in HCC cells after XON9 treatment assessed by MitoSoxRed staining. Mean fluorescence intensities (MFI) are shown (**D**). Data are expressed as mean ± standard error of the mean (SEM). *T*-test * *p* < 0.05; ** *p* < 0.01; *** *p* < 0.001.

The ability of XON9 to induce apoptosis in HCC cell lines was then evaluated. Hep3B, Huh7 and HepG2 cells were exposed to 300 µg/mL of XON9. After 24 h of culture, apoptosis was measured by AnnexinV staining. Primary hepatocytes were not included in these experiments as they could not be kept in culture for more than 4–6 h. Significant upregulation of apoptosis was observed for the three cell lines tested (9.1 ± 2.9% vs. 26.5 ± 4.4% for Hep3B, 8.4 ± 1.3% vs. 32.5 ± 2% for Huh7 and 11.3 ± 0.7% vs. 27.2 ± 4.3% for HepG2) (Figure 2A). Both extrinsic and intrinsic pathways of apoptosis were activated by XON9 as shown by the activation of caspases 8 and 9, respectively (Figure 2B). Depolarization of mitochondrial membrane was observed for the three cell lines (Figure 2C) as well as the production of reactive oxygen species (ROS) (Figure 2D).

### 2.3. Potency Comparaison Between XON9 and Sorafenib

**Figure 3:**
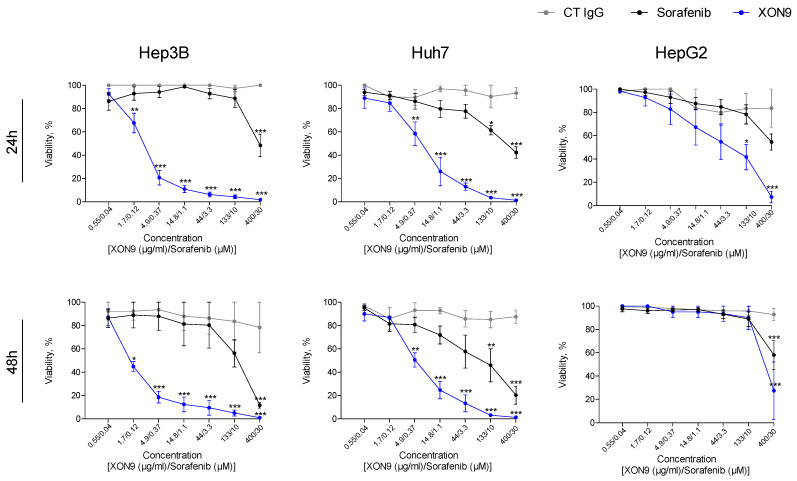
XON9 overall oncolytic activity in 2D cultures. Hep3B, Huh7 and HepG2 cells were treated with XON9 (0.55 to 400 µg/mL) or the standard of care Sorafenib (0.04 to 30 µM) in the presence of 1/6 rabbit serum complement. Cell viability was assessed at 24 h (**top panel**) and 48 h (**bottom panel**) using the CellTiter-Glo viability assay. Two-way ANOVA with Bonferroni post hoc test, * *p* < 0.05; ** *p* < 0.01; *** *p* < 0.001.

As XON9 acts through CDC and apoptosis, we sought to evaluate the combined activity of these two mechanisms against HCC cells. The systemic standard of care drug Sorafenib was used as a comparator. Hep3B, Huh7 or HepG2 cells were treated with increasing concentrations of XON9 or Sorafenib in the presence of rabbit complement, and cell viability was assessed at 24 and 48 h. The highest tested concentration of Sorafenib (30 µM) was chosen to encompass the plasma concentration in patients receiving 400 mg of this drug bi-daily. XON9 showed the strongest lytic activity against the three HCC cell lines tested at 24 and 48 h, with significant lysis of Hep3B or Huh7 from 1.7 µg/mL or 4.9 µg/mL of XON9 treatment (Figure 3). HCC lysis with Sorafenib was effective at the highest concentrations tested only (10 or 30 µM). Activity of Lenvatinib, the other approved multikinase inhibitor for HCC, was comparable to Sorafenib. A slight progression of cell lysis between 24 and 48 h was observed with XON9 for Hep3b (EC50: 2.36 µg/mL vs. 1.4 µg/mL) and Huh7 (EC50: 7.21 µg/mL vs. 4.99 µg/mL). By contrast, the lysis of HepG2 cells was slightly less at 48 h.

**Figure 4:**
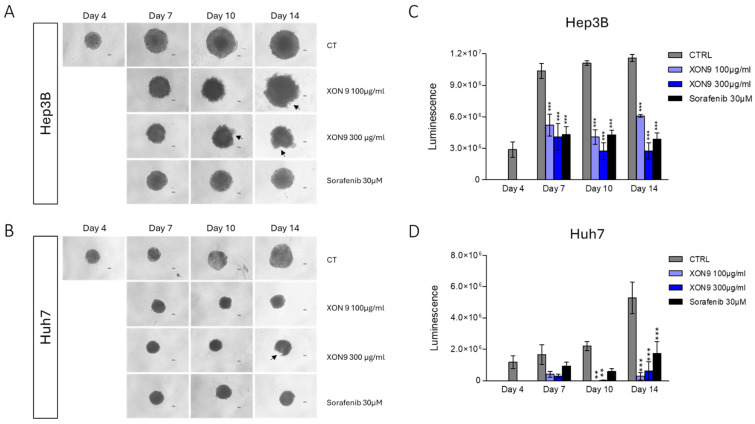
XON9 oncolytic activity on spheroid models. Hep3B or Huh7 spheroids were treated at day 4, 7 and 10 with XON9 (100 or 300 µg/mL) or Sorafenib (30 µM) in the presence of 1/6 rabbit serum complement. Photomicrograph of Hep3B (**A**) or Huh7 (**B**) spheroids at day 4, 7, 10 and 14. Arrows show tumor sphere damages. Cell viability in Hep3B (**C**) or Huh7 (**D**) spheroids determined by the CellTiter-Glo 3D viability assay. Data are expressed as mean ± SEM. Two-way ANOVA with Bonferroni post hoc test, ** *p* < 0.01; *** *p* < 0.001.

To investigate the cytotoxic activity of XON9 in a representative 3D-model, Hep3B and Huh7 cell spheroids were generated by 4-day culture in low attachment plates as already published [20]. Spheroids were then exposed to XON9 or Sorafenib in the presence of rabbit complement for 10 subsequent days. We observed a marked reduction in the Huh7 tumor sphere size at day 10 (~45%) or 14 (~65%), in the XON9 or Sorafenib conditions (Figure 4A,B). Moreover, damages to the Hep3B or the Huh7 sphere periphery were observed on day 10 and 14 after XON9 treatment (Figure 4A,B). Viable cell number within the spheroids was evaluated with a luminescent assay detecting adenosine triphosphate (ATP). XON9 (100 or 300 µg/mL) and to a lesser extent Sorafenib treatments dramatically and significantly reduced the content of viable cells within the spheroids at day 7, 10 and 14 (Figure 4C,D).

### 2.4. Xenograft Mice Model

**Figure 5:**
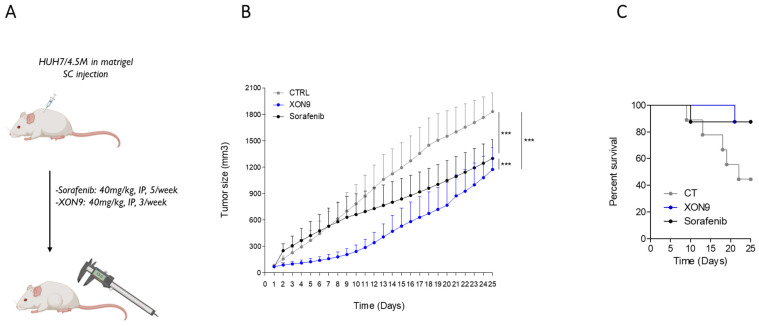
Xenograft mice model. NMRI nude mice were injected subcutaneously (SC) with 4.5 × 10^6^ Huh7 cells. Treatments CT (*n* = 9), XON9 (*n* = 8) or Sorafenib (*n* = 8) were initiated at the onset of tumor growth (approximately 50–100 mm^3^) (**A**). Tumor growth (**B**) was assessed by measuring tumor volume twice weekly for a total of 25 days. Data are expressed as mean ± SEM. Two-way ANOVA, *** *p* < 0.001. Kaplan Meyer curves (**C**).

The anti-tumor activity of XON9 was tested in a xenograft mice model. NMRI nude mice were injected with 4.5 × 10^6^ Huh7 cells before treatment with XON9 or Sorafenib. Treatments were initiated when tumor size reached around 50–100 mm^3^ and were administrated 3 times a week for XON9 and 5 times a week for Sorafenib (Figure 5A). Tumor progression was significantly reduced in the XON9 group compared with the control or Sorafenib groups (36% vs. 29% tumor reduction on day 25 between XON9 and Sorafenib treatments) (Figure 5B). Although not statistically significant, survival on day 25 was higher in the XON9 and Sorafenib groups (87.5%) compared to the control one (44%).

### 2.5. Pharmacokinetic and Safety Parameters

**Figure 6:**
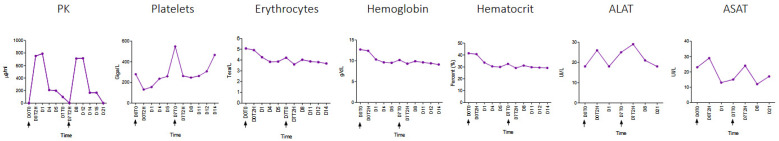
Pharmacokinetics and safety parameters evaluated in a non-human primate. Two intravenous injections (black arrows) of XON9 (50 mg/kg) with a 7-day interval were performed in a female Olive Baboon (*Papio Anubis*). PK, blood factors (platelets, erythrocytes and hemoglobin) and transaminases (ALAT and ASAT) were investigated.

The tolerance and the pharmacokinetic parameters of XON9 in a non-human primate model was then evaluated. Two intravenous injections of XON9 (50 mg/kg) were performed in one Olive Baboon within a 7-day interval. The animal was then monitored for 21 days for blood or clinical parameters. A rapid increase in swine IgG concentration was observed after the first injection of XON9 (Cmax ~ 800 µg/mL obtained at day 1). Then, swine IgG concentration decreased up to the second injection of XON9; no evident accumulation was observed after the second dose compared to the first (Figure 6). A slight decrease in erythrocytes haemoglobin and haematocrit was observed along the observation period. Platelet number dropped (~50%) after XON9 injections but quickly returned to baseline level. A faint and temporary increase in blood aspartate transaminase (ASAT) and alanine transaminase (ALAT) was observed after XON9 injection suggesting a minimal impact on the liver or bile ducts (Figure 6). Throughout the toxicological evaluation conducted, no clinical abnormalities were observed during the study period. Parameters such as body temperature, general behavior, and weight progression remained within normal ranges for the species. Altogether, these findings suggest the absence of toxicity or adverse clinical effects associated with XON9.

## 3. Discussion

HCC is a major health problem with almost 1 million new cases diagnosed each year and nearly as many deaths [4]. Systemic therapies with multikinase inhibitors show modest efficacy in patients generally diagnosed with advanced disease. Immunotherapies with mAb showed promising results. Yet, they apply to a small fraction of patients, and they remain sensitive to tumor escape. New therapeutic approaches should thus be envisaged to improve the management of HCC.

We evaluate here the antitumor activity and the safety of XON9, a first-in-class GH-pAb targeting HCC. XON9 was produced by hyperimmunization of pigs deficient in *CMAH* and *GGTA1* genes with a human liver cell line. XON9 showed the most potent CDC activity against Hep3B and Huh7 while normal hepatocytes were spared at low concentration. Polyclonal Ab are complex preparations recognizing multiple antigens and the molecular basis of pAb reactivity/specificity against tumor or primary cells remains largely unexplored [16,21,22,23]. Nevertheless, specificity of XON9 towards tumor tissues has already been observed [16]. XON9 was also reactive against unrelated tumor cell lines such as HCT116 (colon) or A549 (lung) but not Capan (pancreas) cells. Comparable cross reactivity has been observed as well with the ATG Fresenius rabbit IgG, directed against the Jurkat T-lymphoma cell line, which is able to lyse acute myeloid leukemia or B-cell lymphoma cell lines [24]. Interestingly, the CDC activity of XON9 against HepG2 was lower compared to Hep3B or Huh7 although binding was similar between the three cell lines. Differences in resistance to cell lysis or in the antigens bound by XON9 may explain this variation in sensitivity to XON9. Expression of complement regulators (CD46, CD55 and CD59) was comparable between the three HCC cell lines tested. No release of the soluble active form of CD46 was detected in our cell cultures. Blockade of CD46 and CD59 partially restores CDC activity in HepG2 cells, suggesting a differential inhibitory activity of these molecules depending on the cell lines. Deciphering the repertoire of antigens recognized by XON9 will also be useful to understand the molecular basis of XON9 reactivity.

We then evaluated the ability of XON9 to induce apoptosis in the three cell lines tested. XON9 concentration was set at 300 µg/mL and exposure was prolonged to 24 h to allow the apoptosis process to occur. XON9 induced apoptosis in the three cancer cell lines tested. The comparison with normal primary hepatocytes was not possible as these cells could not be maintained in culture for the 24 h of the apoptosis assay. Apoptosis induction seems to be a hallmark of pAb targeting tumors [16,21,23,25,26,27]. Yet, the mechanisms involved remain elusive. We showed here the activation of both the extrinsic and the intrinsic signalling pathways after XON9 exposure. As XON9 recognized multiple antigens on tumor cells, it probably activated death receptors, leading to the activation of the extrinsic apoptosis pathway. Mitochondria health and activity were impacted as well, as shown by the loss of mitochondrial membrane potential and the production of superoxide radicals. The binding of XON9 to various receptors on cell surface and their uncontrolled activation/inhibition might thus translate to cellular stress and mitochondria dysfunction, eventually leading to the activation of the intrinsic apoptosis pathway. Human protein array analyses identified cellular targets of XON9, such as solute carrier family 3 member 2 (SLC3A2), annexin A5 (ANXA5), fatty acid synthase (FASN) or reticulon 4 (RTN4) involved notably in tumor progression, angiogenesis, cell growth or apoptosis (Supplementary Table S1 [28,29,30,31,32]). Interestingly, among them, SLC3A2 is the target of mAb [33] or CAR-T cell [34] therapies. Further works will be necessary to evaluate the contribution of XON9 targets in the signalling cascade of apoptosis.

The activity of XON9 was then assessed and compared to the standard of care Sorafenib in vitro in 2D-monolayer and 3D-spheroid cultures and in vivo in a xenograft mice model. Overall, XON9 showed a stronger antitumor activity compared to Sorafenib with some nuances depending on the model. Difference in activity between XON9 and Sorafenib is the most marked in 2D monolayers. The short length of drug exposure (24/48 h only for the 2D monolayers) probably reduced Sorafenib activity. The 3D structure might also protect cells from lysis by reducing the diffusion of drugs and complement within the sphere [35]. In vivo, XON9 significantly reduced tumor progression compared to control or to Sorafenib groups. As mouse complement is not active with pig IgG [36], the activity of XON9 observed in the xenograft model depends mostly upon apoptosis, even though other mechanisms such as phagocytosis can be involved [16]. Thus, by targeting multiple epitopes and triggering various mechanisms of action, XON9 increases the probability of tumor response while reducing the risk of immune escape.

The safety and tolerability of GH-pAb have been demonstrated in human in the setting of transplantation or infectious diseases [17,18,19,37]. We sought to investigate the safety of XON9 in an Olive Babon. In vitro experiments showed a specificity of XON9 towards HCC cells. XON9 plasma concentration of 50–100 µg/mL might thus be an acceptable dose, targeting tumor and sparing normal tissues. Injection of 50 mg/kg of XON9 resulted in a plasma concentration of 800 µg/mL, far above the projected active concentration in humans. At this concentration, despite a moderate and brief increase in transaminases, no liver toxicity was observed. Blood parameters remain also unaffected. Although no definite conclusion can be drawn from a unique animal, these data suggest the safety of XON9.

In conclusion, XON9 show promising antitumor activity and an acceptable safety profile. Within the context of multimodal therapy, XON9 could therefore represent a potential therapeutic option to be further explored in the management of refractory HCC.

## 4. Material and Method

### 4.1. Preparation of XON9

Pigs double knocked out for cytidine monophospho-N-acetylneuraminic hydroxylase (*CMAH*) and alpha-1,3-galactosyltransferase (*GGTA1*) were immunized with a human liver cell line. All experimental procedures were approved by the Animal Ethics Committee of the Pays de la Loire and authorized by the French Ministry of Higher Education and Research (APAFIS 35612). Blood collected from hyper-immunized animals was stored at room temperature for 3–5 h. Serum was then separated from clot by centrifugation and IgG were purified by protein A affinity chromatography.

### 4.2. Cells, Culture Media and Reagents

Human HCC cell lines (Hep3B, Huh7 and HepG2) were cultured in Dulbecco’s Modified Eagle Medium (DMEM) (Sigma, Saint-Quentin-Fallavier, France) supplemented with 10% Foetal Calf Serum (FCS) (Biowest, Nuaillé, France). Primary human hepatocytes (Biopredic International, Saint Grégoire, France) were cultured in Basal Hepatic Cell Medium (BHCM) (Biopredic International) according to the manufacturer’s instruction. Rabbit complement was purchased from Cedarlane Labs (Burlington, ON, Canada).

### 4.3. Complement Dependant Cell Cytotoxicity (CDC)

Tumor cells or primary hepatocytes were plated in U bottom 96-well plates and incubated with serial dilution of XON9 with rabbit complement (1/6) in DMEM 10% FCS or BHCM. After 90 min incubation at 37 °C and 5% CO_2_, cells were washed twice and stained with Acridine Orange and 4′,6-diamidino-2-phenylindole (DAPI) (both from Chemometec, Allerod, Denmark). Cell viability was then measured on a NucleoCounter^®^ NC-3000™ Advanced Image Cytometer (Chemometec).

### 4.4. Apoptosis Assay

Tumor cells were cultured at 37 °C and 5% CO_2_ with 300 µg/mL of XON9 in DMEM 10% FCS. After 24 h, cells were collected, washed twice with DMEM 10% FCS and labelled with CF488-conjugated Annexin V (Biotium, Freemont, CA, USA) for 15 min at 37 °C. After being washed twice with DMEM 10% FCS, cells were stained with 10 µg/mL propidium iodide (PI) (Chemometec) before analysis on a Nucleocounter^®^ NC3000™ Advanced Image Cytometer. Percentages of cells in early apoptosis (Annexin V+/PI− cells) and in late apoptosis (Annexin V+/PI+) were combined to determine the overall % of apoptosis.

### 4.5. Mitochondrial Potential Assay

Tumor cells were cultured at 37 °C and 5% CO_2_ with serial dilution of XON9 in DMEM 10% FCS. After 24 h, mitochondrial transmembrane potential was determined with Tetraethyl benzimidazolyl carbocyanine iodide (JC-1) staining. Briefly, cells were collected, washed twice in DMEM 10% FCS and labelled with 2.5 µg/mL JC-1 (Chemometec) in PBS for 15 min at 37 °C. After 2 washes with PBS, cells were stained with 1 µg/mL DAPI (Chemometec) and fluorescence was read on a Nucleocounter^®^ NC3000™ Advanced Image Cytometer. Red-JC-1^high^/Red-JC-1^low^ fluorescence intensity ratio was used as a mitochondrial membrane potential marker.

### 4.6. Reactive Oxygen Species Assay

Tumor cells were cultured at 37° C and 5% CO_2_ with serial dilution of XON9 in DMEM 10% FCS. After 24 h, production of mitochondria superoxide was assessed with the MitoSOX Red probe (MedChemExpress, Monmouth Junction, NJ, USA). Cells were collected, washed twice with PBS and then incubated with 5 µM of MitoSOX Red and 1 µg/mL Hoescht (Chemometec) in PBS for 30 min at room temperature in the dark. After two washed with PBS, fluorescence was read on a Nucleocounter^®^ NC3000™ Advanced Image Cytometer.

### 4.7. Caspases 8 and 9 Assays

Tumor cells were cultured at 37 °C and 5% CO_2_ with serial dilution of XON9 in DMEM 10% FCS. After 24 h, caspase activity was determined with Fluorochrome-Labeled Inhibitor of Caspases (FLICA) assays (Immunochemistry Technologies, Davis, CA, USA) according to the manufacturer’s instructions. Cells were collected, washed twice with DMEM 10% FCS and labeled with FAM-LETD-FMK (caspase 8) or FAM-LEHD-FMK (caspase 9) in DMEM 10% FCS for 1 h at 37 °C. After 2 washes, cells were stained with 10 µg/mL of PI and fluorescence was read on a Nucleocounter^®^ NC3000™ Advanced Image Cytometer. Overall caspase activity was determined by the level of green fluorescence in PI+ or PI− cells.

### 4.8. Cancer Cell Viability

Tumor cells were plated in 96-well plates (U bottom) and incubated with serial dilution of XON9 or Sorafenib (MedChemExpress) in the presence of rabbit complement (1/6) in DMEM 10% FCS. After 24 or 48 h, cells were washed with DMEM 10% FCS and resuspended in CellTiter-Glo (Promega, Charbonnière-les-Bains, France). Luminescence was read after 10 min on a GloMax Navigator microplate luminometer (Promega). For each cell line, a range of cells was prepared to calculate the number of viable cells within each well. For each condition, cell viability was then calculated as below:

$$Viability=100+\left[\left(Number\;of\;cells\;in\;the\;well-Number\;of\;cells\;in\;control\;well\right)\times 100\right]/Number\;of\;cells\;in\;control\;well$$

### 4.9. Tumor Spheroid Assay

Spheroids were prepared from the cancer cells Hep3B and Huh7 according to a protocol previously published [20]. Tumor cells (1000) were seeded in 96-well nuclon sphera 3D low attachment culture plates (Thermo Fisher Scientific, Waltham, MA, USA) in 200 µL DMEM supplement with 10% FCS. Spheroid formation was initiated by centrifugation of the plates at 1000× *g* for 10 min. Repeated treatments with XON9 (100, 300 µg/mL) or Sorafenib (30 µM) were performed at day 4, 7 and 10 in presence of rabbit complement (1/6). Cell viability was measured at day 4, 7, 10 and 14 using the Cell Titer Glo 3D reagent (Promega). Luminescence was read on a GlowMax Navigator microplate luminometer.

### 4.10. Tumor Xenograft Model

A subcutaneous heterotopic tumor model was used with immunodeficient NMRI nude female mice (Janvier-Labs, Le Genest-Saint-Isle, France) according to [16] with some modifications. Mice, 8 to 12-week-old, were maintained at the UTE-IRS Nantes Biotech Animal Facility, with free access to food. Experimental procedures were approved by the Animal Ethics Committee of the Pays de la Loire authorized by the French Ministry of Higher Education and Research (APAFIS 45920). Mice were injected under the skin, in the left dorsal flank with Huh7 (4.5 million in 100 µL of NaCl/Matrigel ratio 1:1). Treatments were initiated when the tumor size reached approximately 50–100 mm^3^. XON9 (40 mg/kg) was administered intraperitoneally three times a week and sorafenib (40 mg/kg) was administered intraperitoneally daily (5/7 days). Tumor growth was monitored for 25 days using a caliper. Tumor volume was calculated using the standard formula: 1/2 × Width × Width × Length, assuming a standard and constant tumor shape.

### 4.11. Pharmacokinetic and Safety Parameters in a Non-Human Primate

Pharmacokinetic and safety parameters were investigated in one female naïve Olive Baboon (*Papio Anubis*), after 2 intravenous injections of XON9 (50 mg/kg each), with a 7-day interval. XON9 (2.5 mg/mL in NaCl) was injected under general anesthesia (2% isoflurane), with continuous monitoring of vital parameters by electrocardiogram and measurement of blood pressure and oxygenation. Blood was collected at days 0 (pre+ post injection), 1, 3, 5, 7 (pre+ post injection), 8, 10, 14 and 21. The animal was monitored for 21 days for clinical signs, hepatic, pancreatic, muscular and cardiac assessment and for blood count. Pharmacokinetics (PK) assays were performed as well. At the end of the protocol, the animal was euthanized and subjected to a full post-mortem macroscopic examination. Experimental procedures were approved by the Animal Ethics Committee of the Pays de la Loire and authorized by the French Ministry of Higher Education and Research (APAFIS 46936).

## Data Availability

Data is contained within the article or Supplementary Material.

## Supplementary Materials

The following supporting information can be downloaded at https://www.mdpi.com/article/10.3390/ijms26189185/s1.
