# Supplementary material for: XON9—A Glyco-Humanized Polyclonal Antibody Effective Against Hepatocellular Carcinoma
Source: Int J Mol Sci. 2025 Sep 20;26(18):9185. doi: 10.3390/ijms26189185 (PMC12470362; doi:10.3390/ijms26189185)
Supplement: Supplementary file 1 [file ijms-26-09185-s001.zip › ijms-3698718-supplementary.pdf]

Supplementary Figure S1. XON9 activity against HCT116, A549 and CAPAN cell lines. CDC activity of XON9 against HCT116 (colon) (n=2), A549 (lung) (n=2) and CAPAN (pancreas) (n=1). XON9 activity against HepG2 cell line was used as a control. Mean ± SEM.

Supplementary Figure S2: XON9 binding to HCC cells**.** Tumor cells were plated in U bottom 96-well plates and incubated with serial dilution of XON9 in DMEM 10% FCS. After 30 minutes incubation at 4°C, cells were washed twice with DMEM 10% FCS. Bound IgG were then detected with AF488-conjugated protein-G (Thermo Fisher Scientific) in DMEM 10% FCS, 30 minutes at 4°C. After two washes, fluorescence was measured on a NucleoCounter® NC-3000™ Advanced Image Cytometer (Chemometec).

Supplementary Figure S3. Recruitment of C1q and expression of complement regulators by HCC cells. (**A**) C1q binding to HCC cells. Hep3B, Huh7 and HepG2 were plated in U bottom 96-well plates and incubated for 1h at 4°C with serial dilution of XON9 in the presence of rabbit complement (1/6) in DMEM 10% FCS. After 2 washes, FITC-conjugated anti-C1q (Biorad, Marnes la Coquettes, France) was added for 30 minutes at 4°C in DMEM 10% FCS and fluorescence was measured after 3 washes on a NucleoCounter® NC-3000™ Advanced Image Cytometer (Chemometec). (**B**) Complement regulator expression by Hep3B, Huh7 and HepG2 cells. Tumor cells were stained for 30m minutes at 4°C with either AF488-conjugated anti-human CD46 (Abcam, Cambridge, UK), AF488-conjugated anti-human CD55 (Thermo Fischer Scientific) or AF647-conjugated anti-human CD59 (Thermo Fischer Scientific) antibodies. After two washes, fluorescence was measured on a NucleoCounter® NC-3000™ Advanced Image Cytometer (Chemometec).

Supplementary Figure S4. XON9 oncolytic activity after CD46 or CD59 blockade. Hep3B, Huh7 and HepG2 cells were treated with 1µg/ml of anti-CD46 (Sino Biological, Eschborn, Germany) or anti-CD59 (Abnova, Taoyuan City, Taiwan) for 30min before incubation with XON9 in the presence of 1/6 rabbit serum complement. Cell viability was assessed at 24h using the CellTiter-Glo viability assay (n=2).

Supplementary Figure S1

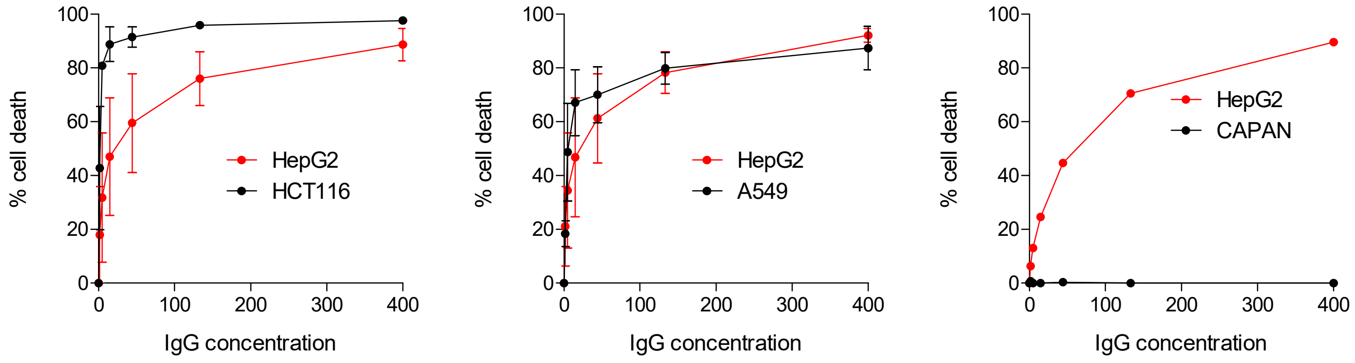

Supplementary Figure S2.

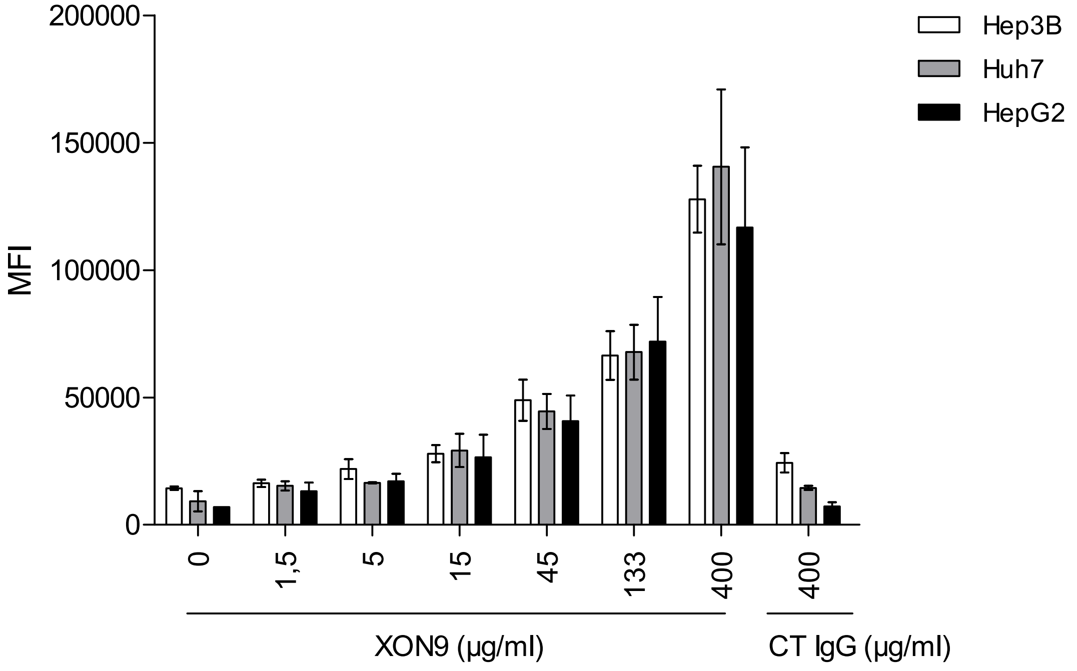

Supplementary Figure S3.

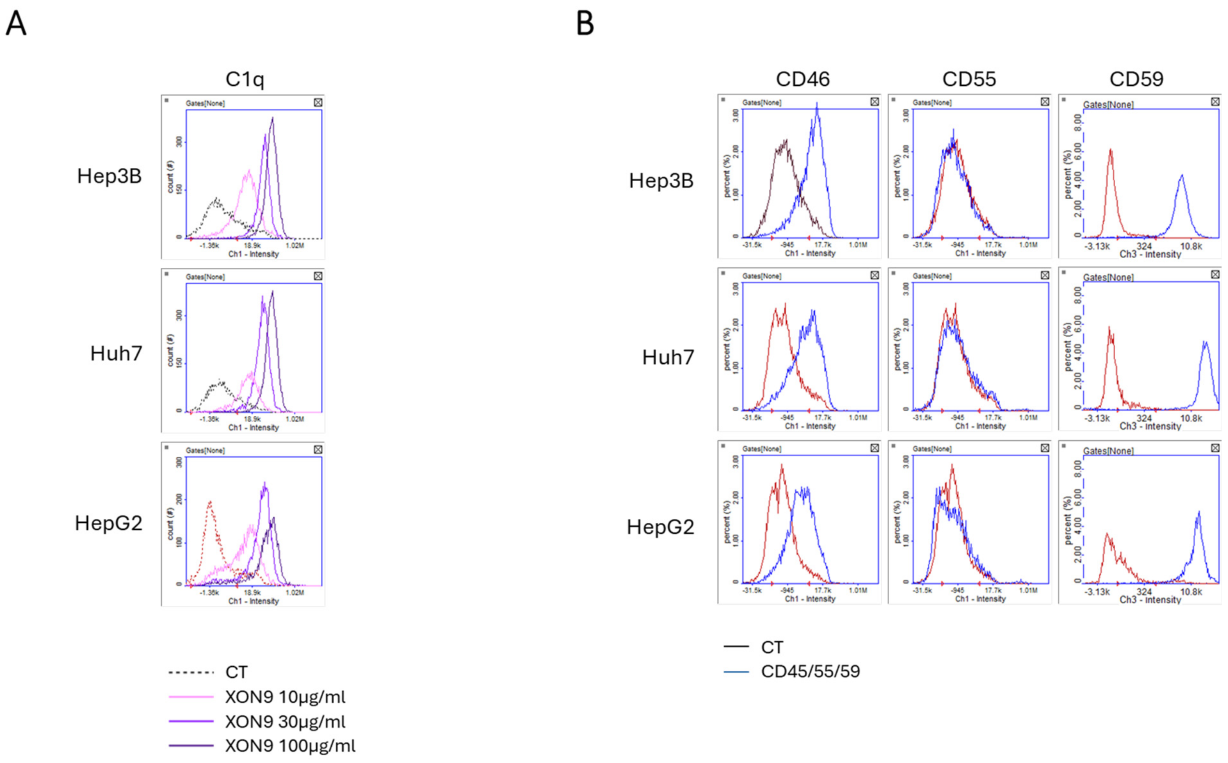

Supplementary Figure S4.

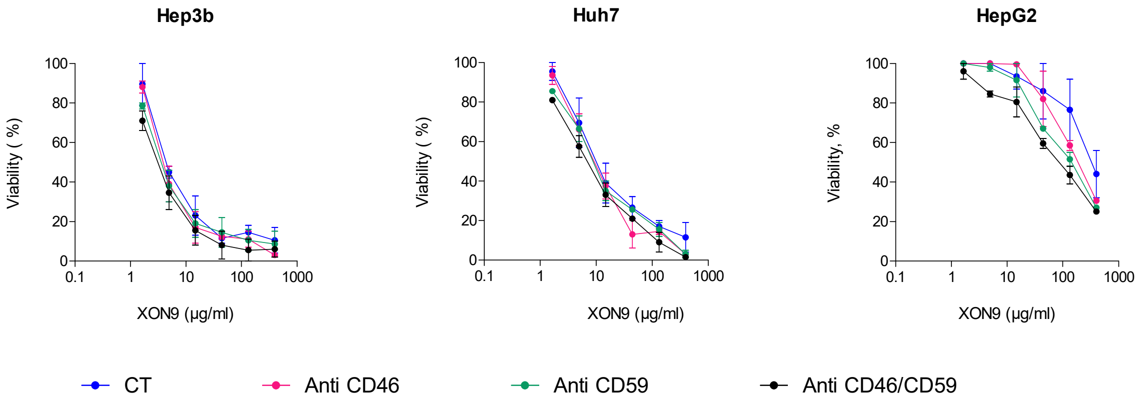

Supplementary Table S1.

Protein	Z score	Role	Ref
SLC3A2	81.5	Tumor progression, angiogenesis, proliferation	[1]
ANXA5	56.8	Apoptosis, immunosuppression	[2]
FASN	31	Cell growth	[3]
RTN4	26.8	Cell growth, metastasis, angiogenesis	[4, 5]

Supplementary Table S1. Targets (non-exhaustive) of XON9 identified by human protein arrays and their respective role in HCC or tumor development.

1. Canup, B.S.B.; Song, H.; Laroui, H. Role of CD98 in Liver Disease. *Ann Hepatol* 2020, *19*, 602–607.
2. Jin, M.; Zhang, J.; Sun, Y.; Liu, G.; Wei, X. ANXA5: Related Mechanisms of Osteogenesis and Additional Biological Functions. *Front Cell Dev Biol* 2025, *13*.
3. Che, L.; Paliogiannis, P.; Cigliano, A.; Pilo, M.G.; Chen, X.; Calvisi, D.F. Pathogenetic, Prognostic, and Therapeutic Role of Fatty Acid Synthase in Human Hepatocellular Carcinoma. *Front Oncol* 2019, *9*.
4. Cai, H.; Saiyin, H.; Liu, X.; Han, D.; Ji, G.; Qin, B.; Zuo, J.; Shen, S.; Yu, W.; Wu, J.; et al. Nogo-B Promotes Tumor Angiogenesis and Provides a Potential Therapeutic Target in Hepatocellular Carcinoma. *Mol Oncol* 2018, *12*, 2042–2054, doi:10.1002/1878-0261.12358.
5. Zhu, B.; Chen, S.; Hu, X.; Jin, X.; Le, Y.; Cao, L.; Yuan, Z.; Lin, Z.; Jiang, S.; Sun, L.; et al. Knockout of the Nogo-B Gene Attenuates Tumor Growth and Metastasis in Hepatocellular Carcinoma. *Neoplasia (United States)* 2017, *19*, 583–593, doi:10.1016/j.neo.2017.02.007.
